# Genealogie, Züchtung und (Bio‑)Politik. Wissensgeschichtliche Analysen zur Formierung des Rassendiskurses in der Frühen Neuzeit und Moderne

**DOI:** 10.1007/s00048-026-00452-w

**Published:** 2026-09-17

**Authors:** Susanne Lettow

**Affiliations:** Margherita-von-Brentano-Zentrum für Geschlechterforschung, Freie Universität Berlin, Habelschwerdter Allee 30, 14195 Berlin, Deutschland

**Keywords:** Genealogy, Breeding, Race discourse, Biopolitics, Genealogie, Züchtung, Rassendiskurs, Biopolitik

Mackenzie Cooley 2022. *The Perfection of Nature. Animals, Breeding and Race in the Renaissance.* Chicago, IL: The University of Chicago Press, brosch., 344 S., 30 Abb., 37,50 US$, ISBN: 978-0-226-82228‑0.

William Max Nelson 2024. *Enlightenment Biopolitics. A History of Race, Eugenics, and the Making of Citizens.* Chicago, IL: The University of Chicago Press, brosch., 328 S., 10 Abb., 35,00 US$, ISBN: 978-0-226-82558‑8.

Stefani Engelstein 2024. *Geschwister-Logik. Genealogisches Denken in der Literatur und den Wissenschaften der Moderne*. Berlin: DeGruyter, geb., 325 S., 17 Abb., 109,95 €, ISBN: 978-3-111-24741‑0.

Marianne Sommer 2024. *The Diagrammatics of* ‚*Race*‘*. Visualizing Human Relatedness in the History of Physical, Evolutionary, and Genetic Anthropology, ca. 1770–2020*. Cambridge: Open Book Publishers, brosch., 372 S., 92 Abb., Open Access, 31,95 £, ISBN: 978-1-80511-260‑0.

Die Auseinandersetzung mit der Geschichte des modernen Rassendiskurses, d.h. mit der Gesamtheit von sprachlichen und nicht-sprachlichen Praktiken, ihren strukturellen Bedingungen und materiellen Auswirkungen, die das Konstrukt „Rasse“ hervorgebracht haben, sowie mit den Verschränkungen dieses Diskurses mit Konzeptionen von Herkunft und Zugehörigkeit, Züchtung und Selektion hat in den vergangenen zwanzig Jahren durch die Erforschung der Geschichte der Vererbung und Reproduktion wichtige Impulse erhalten (u. a. Weinbaum 2004, Müller-Wille & Rheinberger 2007; Figal 2010; Stephanson & Wagner 2015; Brandt & Müller-Wille 2016; Hopwood et al. 2018). Dabei haben diese Forschungen verdeutlicht, dass sich mit der Entwicklung der Wissenschaften vom Leben ab der zweiten Hälfte des 18. Jahrhunderts Konzepte von Fortpflanzung, Abstammung und Verwandtschaft herausbildeten, die mit der kolonialen Expansion Europas ebenso verflochten waren wie mit der Transformation von Familienstrukturen (vgl. Müller-Wille 2015, 2022; Mazzolini 2007; Sabean et al. 2007). Die im Folgenden zu besprechenden Arbeiten erweitern diese Ansätze.

## Zwischen Veränderung und Fixierung: Züchtungspraktiken und -vorstellungen in der Renaissance

Mackenzie Cooleys Untersuchung von Züchtungspraktiken und -vorstellungen in der Renaissance stellt zwei bislang unterbelichtete Aspekte ins Zentrum: zum einen das Verhältnis von Tier- und Menschenzucht und zum anderen die Bedeutung der kolonialen Eroberung der Amerikas und des sogenannten „Columbian Exchange“ (Crosby 1972). Durch einen vom „Animal Turn“ der Kultur- und Geisteswissenschaften inspirierten Blick sowie die Einbeziehung von Dokumenten indigener, mesoamerikanischer Züchtungspraktiken und -vorstellungen leistet sie einen innovativen Beitrag zum Verständnis der Formierung des Konzepts der *limpieza de sangre* – das heißt einer Vorstellung von „Reinheit des Blutes“, die zunächst durch die Inquisition formuliert und im rassistisch-genealogischen Denken der Moderne immer wieder reartikuliert wurde. Der Titel des Buches *The Perfection of Nature* verweist auf den Jesuiten José de Acosta, der über fünfzehn Jahre in den süd- und mittelamerikanischen Kolonien verbrachte und nach seiner Rückkehr nach Spanien eine *Historia natural y moral de las Indias* (1590) verfasste, in der er eine umfassende Darstellung der „Neuen Welt“ lieferte. Acosta ging von einer universellen Perfektionierbarkeit aller einstmals auf Noahs Arche versammelten Tiere – inklusive des Menschen – aus. Diese Annahme brachte ihn Cooley zufolge in Konflikt mit der Inquisition, da sie die Möglichkeit der religiösen Konversion zu untermauern und die Vorstellung von physisch (im „Blut“) verankerten mentalen und spirituellen Fähigkeiten und Eigenschaften zu widerlegen schien. Cooley orientiert sich in ihrer Analyse an dem etwas schematischen Gegensatz von Variabilität und Fixierung körperlicher und nicht-körperlicher Merkmale, der in der Anspielung auf Acosta im Titel des Buches angedeutet wird. Dieser Gegensatz erlaubt es ihr, die ansonsten weitgehend unverbundenen Untersuchungen zu Züchtungspraktiken und -vorstellungen im 16. Jahrhundert zumindest lose zueinander in Beziehung zu setzen.

Die Struktur des Buches ist, wie Cooley unter Rückgriff auf jene musikalische Analogie erläutert, die Edward Said bereits in *Culture and Imperialism* (1993) herstellte, kontrapunktisch. In vier Teilen werden in jeweils zwei Kapiteln gegenläufige Züchtungspraktiken und -vorstellungen dargestellt. Im ersten Teil widmet sich Cooley zunächst dem praktischen, insbesondere auf die Pferdezucht bezogenen Wissen. Im Anschluss diskutiert sie die Praxis des Brandmarkens, die das Ziel verfolgte, die Zugehörigkeit zu einer *razza *oder einem Fürstenhaus zu fixieren – trotz und wegen der Tatsache, dass die gezüchteten Merkmale meist höchst instabil waren. Teil zwei rekonstruiert Züchtungspraktiken am Hof der Gonzaga in Mantua, für die sich im 19. Jahrhundert schon Jacob Burckhardt interessierte, der die These vertrat, dass hier Grundlagen der wissenschaftlichen Zoologie und Botanik gelegt wurden (15). Allerdings ging es in Mantua nicht nur um Pflanzen- und Tierzucht, wie Cooley mit Rekurs auf Isabella d’Estes Züchtung kleinwüchsiger Menschen zeigt. Kontrastierend dazu untersucht Cooley Sammel- und Züchtungspraktiken am mexikanischen Hof von Moctezuma sowie das für mexikanische Züchtungspraktiken zentrale Konzept *xinachtli – *„linking maize cultivation, semen and brotherhood“ (16). Sie greift dabei auf Wörterbücher, Chroniken und den *Florentine Codex* (1580) zurück – einem unter Beteiligung von Nahua-Künstler:innen erstellten Kompendium, in dem nach der spanischen Eroberung indigene soziale Praktiken sowie Wissen über Pflanzen und Tiere festgehalten wurden. In Teil drei beschäftigt sich Cooley mit der Tierwelt in Mittel- und Südamerika nach der kolonialen Eroberung und behandelt zum einen die durch *mestizaje *entstandenen neuen Hundearten sowie die Wahrnehmung von Lamas und Alpakas durch die europäischen Kolonisator:innen als friedliche „christliche“ Schafe oder aber feindliche „muslimische“ Kamele. Dabei geht es ihr um eine Ergänzung der Forschungen zum sogenannten „Columbian Exchange“ (Crosby 1972) – das heißt den ökologischen und epidemiologischen Implikationen der Zirkulation von Pflanzen und Tieren infolge der kolonialen Eroberung der Amerikas –, indem sie zum einen die Entstehung neuer Arten und „Mischungen“, zum anderen die Bemühungen, die Tiere in den Amerikas entlang europäischer Denkmuster einzuteilen, hervorhebt.

Der vierte und letzte Teil analysiert die Schriften von zwei europäischen Autoren, die in Bezug auf die Frage der Variabilität oder Fixierung physischer und mentaler Eigenschaften unterschiedliche Positionen vertraten, um hier erneut zu zeigen, dass diese Tendenzen zeitlich koexistierten. Cooley kontrastiert José de Acostas Vorstellungen einer flexiblen, perfektionierbaren Natur mit Giovanni Battista della Portas Projekt einer Physiognomie, durch die verborgene Eigenschaften anhand äußerlicher Körpermerkmale entziffert werden sollten.

Insgesamt ist Cooleys Analyse in Hinblick auf die Kontrastierung europäischer und indigener Praktiken und Konzepte innovativ und bringt eine Reihe spannender Einsichten hinsichtlich der Bedeutung von Tier- und -pflanzenzüchtung für die Formierung des modernen Rassendiskurses. Allerdings werden historische, kulturelle und epistemische Bezüge zwischen den Fallstudien eher evoziert als expliziert und die Differenz Fixierung/Variablität verbindet die einzelnen Analysen nur auf einer sehr abstrakten Ebene. Zudem deutet Cooley zwar eine lange historische Kontinuität der von ihr untersuchten Problematik an, wenn sie ihre Untersuchung im Rahmen einer Geschichte der Eugenik verortet und mit dem Ziel verbindet, „to redefine a longer history of a practice of which nineteenth- and twentieth-century scientific eugenics was only a small part“ (4). Ihre Analysen zur Verschränkung zwischen europäischen und mesoamerikanischen, auf Tiere und Menschen bezogenen Züchtungspraktiken und -vorstellungen in der Renaissance aber bearbeiten die Frage der so postulierten *longue durée* nicht. Auch eine systematische Auswertung der Einzelanalysen mit Blick auf die Formierung des modernen Rassendiskurses bleibt leider aus.

## Bevölkerungspolitische Kalkulationen und Züchtungsprojekte in der Aufklärung

Auch William Max Nelsons Untersuchung zu den Anfängen der modernen Biopolitik in der Aufklärung setzt sich mit dem Zusammenhang von Züchtungsprojekten und Rassenkonstruktionen auseinander. Mit dem Begriff der Biopolitik knüpft er explizit an Foucault an, möchte aber über diesen hinausgehend zeigen, „that biopolitics emerged as eugenic plans and new ways of dividing up the population“ (2). Diese Abgrenzung ist nicht ganz trennscharf, denn schon Foucault hat den Begriff der Biopolitik explizit mit der These einer rassistischen Fragmentierung der Bevölkerung verbunden und ihn im ersten Band von *Sexualität und Wahrheit* im Kontext seiner Analyse des „Sexualitätsdispositivs“ eingeführt. Zu dessen zentralen Achsen gehört dabei auch „die Sozialisierung der Fortpflanzung“, welche an der politisch-epistemischen Figur des „malthusianischen Paars“ ansetzt (Foucault 1983, vgl. Deutscher 2017). Die Bedeutung von Sexualität, die für Foucault zentral ist, blendet Nelson in seinem Verständnis von Biopolitik jedoch vollkommen aus, und auch sonst bleibt eine tiefergehende Auseinandersetzung mit Foucault aus. Nelson definiert Biopolitik durch zwei Aspekte: den Ausschluss bestimmter Gruppen aus einem „sozialen, politischen oder Gattungskörper“ (3) und das Vorhaben, die im Inneren des Kollektivkörpers verbleibenden Individuen zu transformieren (ebd.). In diesem Zusammenhang spricht er vom Paradox der „malleable equality“ (16) – das heißt einer Gleichheit der Formbarkeit der Körper, die neue Formen von Ungleichheit begründet. Auf Basis dieser Definition von Biopolitik rekonstruieren die sieben Kapitel des Buches die Formierung von Konzeptionen der Menschenzüchtung im 18. Jahrhundert mit Blick auf Frankreich und dessen transatlantische Kolonien.

Im ersten Kapitel stellt Nelson, entsprechend der ideengeschichtlichen Ausrichtung des Buches, die Entstehung eines „organischen Weltbildes“ (18) vor, demzufolge die Natur wie auch der einzelne Körper ein dynamisches, aus einzelnen Elementen zusammengesetztes Ganzes sei. Er greift hier auf einschlägige Forschungen zur Temporalisierung der Natur im Kontext der Formierung der Wissenschaften vom Leben zurück und argumentiert, dass das neue Verständnis „organisierter Körper“ mit einer Rekonzeptualisierung des Verhältnisses von Teil und Ganzem einherging. Dadurch, so Nelson, wurde die aktive Gestaltung des Körpers durch menschliche Intervention präfiguriert.

Das zweite Kapitel stellt die bevölkerungspolitischen Kalküle und Züchtungsprojekte von Autoren wie Charles-Augustin Vandermonde, Maupertuis und Buffon und weniger prominenter Autoren wie Joachim Faiguet de Villeneuve oder, im anglophonen Kontext, John Gregory und Samuel Stanhope Smith vor. Ergänzt wird diese Darstellung durch die Rekonstruktion der in der bisherigen Forschung kaum beachteten politisch-konzeptionellen Entwürfe von Gabriel de Bory und Michel René Hilliard d’Auberteuil – zwei in die Administration der französischen Kolonien eingebundenen Autoren. In diesen Arbeiten, so Nelson, wurden erstmals groß angelegte, bevölkerungspolitisch ausgerichtete Züchtungsprojekte formuliert, die das Ziel verfolgten, die Bevölkerung der Kolonien im Sinne Frankreichs zu optimieren.

Dass solche Überlegungen nicht nur in Bezug auf Bevölkerungen in den Kolonien, sondern auch in Hinblick auf die Zusammensetzung der Bevölkerung innerhalb Frankreichs angestellt wurden, zeigt Nelson im dritten Kapitel. Allerdings geht es hier weniger um Züchtungsprojekte als um Prozesse des „inneren Ausschlusses“ – das heißt der Konstitution von Personengruppen, die zwar in der Gesellschaft leben, aber nicht als vollwertige Mitglieder gelten. Nelson geht in diesem Zusammenhang ausführlich auf Pläne zur Kasernierung von „Vagabunden“ sowie auf die *Police des Noirs *ein – also jene Gesetze, mit denen der Aufenthalt versklavter Personen innerhalb Frankreichs reguliert wurde. Frauen werden als dritte Gruppe behandelt, doch wird nicht deutlich, warum deren Ausschluss aus zentralen gesellschaftlichen Bereichen als Effekt von aufklärerischer Biopolitik behandelt wird. Im Gegenteil reduziert die Homologie von Schwarzen, Vagabunden und Frauen die Frage, welche Rolle die Veränderung von Familien- und Verwandtschaftsformen beziehungsweise der Verschiebung vom Allianz- zum Sexualitätsdispositiv im Kontext der Entstehung biopolitischer Strategien in der Aufklärung spielte, auf die eigentlich banale Feststellung, dass Frauen nicht als gleichwertige Mitglieder der Gesellschaft betrachtet wurden.

Die folgenden zwei Kapitel sind der Zeit der Französischen Revolution gewidmet. Spannend ist dabei insbesondere Nelsons Rekonstruktion von Emmauel-Joseph Sieyès’ biopolitischem Denken und dessen Unterscheidung von „aktiven“ und „passiven“ Staatsbürger:innen im Anschluss an Le Trosnes Konzept von Menschen, „who ‚live amid in society whitout being members of it‘“ (18). Sieyès’ Idee, eine zwischen Mensch und Tier angesiedelte neue Gattung von Lebewesen zu züchten, „capable of serving man for consumption and production“ (Sieyès, zit. n. Nelson: 1), wurde in der politischen Ideengeschichte bisher nur am Rande beachtet. Nelson stellt sie jedoch ins Zentrum von Sieyès’ politischem Denken und in den Kontext seiner Rezeption der zeitgenössischen Naturforschung. Der Ausrichtung des Buches an der politischen Ideengeschichte der Aufklärung folgend widmet Nelson sich im nächsten Kapitel den Überlegungen von Condorcet und Cabanis. Er interessiert sich dabei für deren Verständnis von Gewohnheit als Medium der Regulation physischer und psychischer Prozesse und argumentiert, dass Lamarck auf diese biopolitische Konzeption von Gewohnheit zurückgreifen konnte, als er die These von der Vererbbarkeit erworbener Merkmale formulierte. Zusammenfassend lässt sich festhalten, dass Nelson eine wissenschaftshistorisch informierte politische Ideengeschichte gelungen ist, deren einzelne Bestandteile nicht immer neu sind, die jedoch den jeweiligen Forschungsstand umfassend aufgreift und durch die Zusammenführung unterschiedlicher bevölkerungspolitisch ausgerichteter Züchtungs- und Optimierungsprojekte das Verständnis für die Entstehung der modernen Biopolitik erweitert.

## Genealogisches Denken in der Moderne

Im Gegensatz zu den anderen hier besprochenen Büchern stellt Stefani Engelstein in ihrer Studie zur Struktur genealogischen Denkens in der Moderne den Rassendiskurs zwar nicht ins Zentrum, leistet aber dennoch einen wesentlichen Beitrag zu einer kritischen Rekonstruktion seiner Entstehung. Engelstein analysiert in ihrer im Original bereits 2017 erschienenen Untersuchung das genealogische Denken, das – so die Leitthese – sich im langen 19. Jahrhundert herausgebildet hat und durch eine – wie Engelstein es nennt – „Geschwister-Logik“ strukturiert wird. Die Figur der Geschwister bezieht sich dabei nicht nur auf Personen beziehungsweise literarische Darstellungen von Geschwistern, sondern auch auf epistemische Konstellationen, in denen „Einzelelemente durch die Differenzierung ähnlicher Elemente geschaffen [werden], die daraufhin Geschwisterelemente werden“ (23). Die durch solche Differenzierung produzierten Bestimmungen sind Engelstein zufolge immer schon brüchig, denn das Geschwisterteil „ist ambig – es ist nicht das Selbst und auch nicht ein ganz Anderes“ (2). Es steht daher für „die Kontingenz und Durchlässigkeit von Grenzen, zieht die Ganzheit in Zweifel und hinterfragt die Idee der Einzigartigkeit“ (ebd.). Dieses weit gefasste Verständnis erlaubt es Engelstein in so unterschiedlichen Wissensfeldern wie der Politischen Ökonomie, der Philologie, der physischen Anthropologie, der Evolutionstheorie sowie der vergleichenden Religionswissenschaft und den Rassentheorien des 19. Jahrhunderts eine verbindende „Logik“ zu finden. Sie zeigt dabei die Komplexität jenes Wandels auf, den Foucault als Ablösung der klassischen, auf Ähnlichkeiten ausgerichteten durch eine auf Tiefenstrukturen fokussierende Episteme beschrieb. Auch für die genealogisch angelegten Wissenssysteme der Moderne, so Engelstein, sei nämlich die Analyse von Ähnlichkeiten zentral (3). Indem diese auf Vererbung zurückgeführt werden (ebd.), begründen sie aber nicht mehr in erster Linie Analogien, sondern gelten als Ausdruck von zeitlich artikulierten Abstammungsverhältnissen.

Engelstein geht in zwei weiteren Aspekten über Foucaults Analyse der modernen Episteme hinaus. Erstens macht sie deutlich, dass das moderne genealogische Denken als Teil jener gesellschaftlichen Prozesse begriffen werden muss, die zur Entstehung der bürgerlichen Gesellschaft und ihrer (geschlechter-)politischen Ordnung geführt haben. Dabei knüpft sie an geschichtswissenschaftliche Forschungen zu Familie und Verwandtschaft an, welche die These von der schwindenden Bedeutung verwandtschaftlicher Netzwerke zugunsten der sogenannten Kernfamilie widerlegt haben (u. a. Sabean 1998). Zweitens zeigt Engelstein die Verschränkungen von Episteme, Politik und Affekt der Geschwister-Logik auf. Sie interessiert sich dabei insbesondere für Abwehrstrategien, die durch „das defensive Bestreben, Grenzen zu kontrollieren und zu beherrschen, als Reaktion auf den Schrecken der epistemologischen Instabilität“ (9) gekennzeichnet sind. In besonders drastischer Form sind diese Abwehrstrategien ihr zufolge in den Rassentheorien des 19. Jahrhunderts zum Ausdruck gekommen. Diese werden hier als genealogische Konstruktionen behandelt und in den Kontext der sozialen Transformation und kultureller Aushandlungsprozesse der politischen Bedeutung von Verwandtschaft/Nicht-Verwandtschaft gestellt.

Die Herausforderung, aus der Vielfalt an Wissensformen und Materialien – Engelstein bearbeitet neben wissenschaftlichen, philosophischen und anthropologischen Texten eine Vielzahl literarischer Texte – eine durchgängige epistemische Struktur herauszupräparieren, meistert sie auf überzeugende Weise. Die Analyse wirkt nirgends reduktionistisch, sondern lässt der Spezifik und den Eigenlogiken der untersuchten Texte Raum. Sie vermeidet den Rekurs auf eine lineare Geschichtskonstruktion, auch wenn in der abschließenden Auseinandersetzung mit dem Antisemitismus von Richard Wagner und Paul de Lagarde die Relevanz der Analyse für das Verständnis des genozidalen Antisemitismus des NS-Regimes und die noch immer unabgeschlossene Geschichte von Rassismus und Antisemitismus deutlich wird. Besonders positiv hervorzuheben ist, dass Engelsteins Analyse des genealogischen Denkens auch die geschlechterpolitischen Implikationen der „Geschwister-Logik“ herausarbeitet, die sich zum Beispiel im antisemitisch/rassistischen, sexuell aufgeladenen Phantasma der vom Fremden bedrohten Schwester zeigen, in emanzipatorischer Hinsicht aber vor allem in der – in Literatur, Psychoanalyse und Philosophie lange vernachlässigten – Figur der Ismene. Im Gegensatz zu ihrer Schwester Antigone entzieht sich Ismene der heroischen Tat; in ihrer Familie ist sie die Einzige, der es gelingt, „ihre Hände nicht als Werkzeug für Mord und Suizid einzusetzen“ (67) und die vielleicht für eine Politik steht, „die Dichotomien zugunsten von Differenzen verwirft“ (67). Ganz offensichtlich geht es Engelstein mit ihrer Analyse nicht allein um eine „Genealogie der genealogischen Methoden“ (6) in den genannten Wissensfeldern, sondern auch darum, das „theoretische Potenzial der Geschwister-Logik“ (3) für eine Kritik der Gegenwart freizulegen, die gegen identitäre und ethnonationalistische Anrufungen die Fragilität und Ambiguität des Geschwisterseins verteidigt und praktisch werden lässt.

## Jenseits von Baum und Abstammung? Visualisierungen menschlicher Vielfalt vom 18. Jahrhundert bis zur Gegenwart

Marianne Sommers Buch ist einer Diagrammatik grafischer Darstellungen von „Rassen“ gewidmet– das heißt den bildlichen Visualisierungen menschlicher Diversität von der physischen Anthropologie des späten 18. und frühen 19. Jahrhunderts bis zur Gegenwart, in der seit der sogenannten „ancient DNA revolution“ (7) nicht mehr das Bestreben, ganze, vermeintlich homogene Bevölkerungsgruppen zu klassifizieren, sondern die Analyse von paleoontologischen Prozessen der genetischen Mischung (*genetic admixture*) im Vordergrund steht. Sommer schließt mit ihrer Untersuchung an den „diagrammatic turn“ in den Geistes- und Kulturwissenschaften an, der die epistemischen Funktionen des Einsatzes von Schaubildern ins Zentrum der Aufmerksamkeit gerückt hat. Wie Sommer betont, handelt es sich bei Diagrammen um bildliche Abstraktionen, die darauf zielen, Relationen innerhalb eines bestimmten Gegenstandsbereichs intelligibel zu machen. Sie expliziert dies unter Rekurs auf Charles Sanders Peirce (13), hätte hier aber auch gut an die neueren Debatten um Bildlichkeit und Diagrammatik (vgl. u. a. Heßler& Mersch 2015) anknüpfen können. Das Konzept der operativen Bildlichkeit beispielsweise benennt den für Sommers Analyse so zentralen performativen Charakter von Diagrammen sehr deutlich, wird aber nicht aufgegriffen. Das Buch, das auf Forschungen im interdisziplinären, vom Schweizerischen Nationalfonds (SNF) geförderten Verbundprojekt *In the Shadow of the Tree. The Diagrammatics of Relatedness as Scientific, Scholarly and Popular Practice *zurückgeht, in dem Marianne Sommer, Simon Teuscher, Staffan Müller-Wille und Caroline Arni jeweils Projekte leiteten, behandelt insgesamt die durch den *diagrammatic turn* aufgeworfenen theoretischen und methodischen Fragen nur am Rande. Es präsentiert vielmehr eine detaillierte Geschichte der unterschiedlichen Strategien der Visualisierung von menschlichen Bevölkerungen und menschlicher Vielfalt, die seit dem späten 18. Jahrhundert im Rahmen des modernen Rassendiskurses formuliert wurden.

Sommer geht in ihrer Darstellung chronologisch vor und widmet sich zuerst der Entstehung einer „decidedly diagrammatic traditon“ (4) in der Physischen Anthropologie des 18. Jahrhunderts, zu der Johann Friedrich Blumenbach, Petrus Camper oder Samuel George Morton beigetragen haben. Deren grafische Darstellungen unterschieden sich, wie Sommer zeigt, erheblich mit Blick auf die Frage, ob sie vornehmlich Affinitäten oder Hierarchien zwischen Bevölkerungen beziehungsweise den sogenannten Menschenrassen inszenierten – also entweder im Sinne monogenetischer Argumentationen eine umfassende Verwandtschaft aller Menschen postulierten oder diese im Sinne polygenetischer Positionen vehement bestritten. Interessant ist, dass in diesem Kontext das Baum-Diagramm, das in den folgenden Kapiteln eine zentrale Rolle spielt, noch nicht zum Einsatz kam, obwohl es – so Sommer – bis auf Darstellungen der „menschlichen Familie“ in der christlichen Kosmologie zurückgeht (5). Die erste baumförmige Darstellung von menschheitlichen Verwandtschaftsverhältnissen findet Sommer in dem 1857 von der US-amerikanischen Künstlerin Anna Marie Redfield angefertigten Bild *A General View of the Animal Kingdom*. Für Sommer dient es als Beleg dafür, dass das Baum-Diagramm, das im frühen Rassendiskurs nicht präsent war, dennoch nicht erst mit der Evolutionstheorie in die Forschung zu „human relatedness“ Einzug hielt.

Die Evolutionstheorie steht im Zentrum des zweiten Teils des Buches, in dem Sommer zeigt, dass Darwin selbst mit baumförmigen Darstellungen intensiv experimentierte, erst Haeckel aber das Baum-Diagramm für eine rassistische, polygenetische Darstellung der unterschiedlichen menschlichen Bevölkerungen nutzte. Der dritte Teil des Buches beschäftigt sich mit rassistischen „Radikalisierungen“ dieses hierarchisch-genealogischen Denkens in der ersten Hälfte des 20. Jahrhunderts sowie mit Versuchen, das Baum-Diagramm zu dekonstruieren. Sommer verweist dabei auf ein strukturelles Problem: Baum-Diagramme haben die Tendenz, „to minimize variability within, and maximize distance between human groups“ (6) und auch in der Populationsgenetik sowie in neueren Forschungen, die primär auf die Analyse genetischer Vermischungen ausgerichtet sind, bleiben die epistemische Figur des Baumes und das Problem der Reifizierung, Homogenisierung und Hierarchisierung von Unterschieden zwischen menschlichen Gruppen und Bevölkerungen implizit präsent. Wir sind, so lässt sich auch aus Sommers Analyse schließen, dem genealogischen, am Baum orientierten Denken der Moderne – inklusive der in diesem Denken virulenten Gewalt – noch immer nicht entkommen.

Durchgängig arbeitet Sommer nicht nur die Vermittlungen zwischen Bild und Text, sondern auch die zwischen Denken und Handeln heraus. Sie rekonstruiert, wie die unterschiedlichen Diagramme jeweils in textuell verfassten wissenschaftlichen und politisch-ethischen Argumentationen und Narrativen verortet sind und zeigt, wie diese wiederum mit den „gewaltvollen Praktiken“ (4) von Imperialismus, Kolonialismus, Rassismus und Genozid verschränkt waren. Eine große Stärke des Buches liegt darin, dass Sommer immer wieder auch die kritischen und gegenläufigen Stimmen – zum Beispiel in der Zeit zwischen den Weltkriegen die von Julian Huxley und Franz Weidenreich – zu Wort kommen lässt und deutlich macht, dass das wissenschaftliche Feld der Erforschung und Darstellung menschlicher Diversität heterogene Positionen umfasste, die sowohl in wissenschaftlicher als auch politisch-ethischer Hinsicht umkämpft waren. Insgesamt ist es folgerichtig, wenn Sommer am Ende ihrer historischen Rekonstruktion die seit den 2000er Jahren in der Forschung zu menschlicher Diversität zunehmenden Bilder von Gitter, Geflecht, Fluss oder Netz skeptisch betrachtet – zum einen, weil sie hier vielfach noch immer Bezugnahmen auf die dem 18. und 19. Jahrhundert entlehnte Vorstellung von ursprünglich homogenen Bevölkerungen erkennt, zum anderen, weil wir angesichts der langen Geschichte des Biologismus in Bezug auf eine politisch-ethische Aufladung von biologischem Wissen und biologischen Begriffen ohnehin höchst vorsichtig sein sollten (287). Trotz aller Sympathie, die Sommer für die rhizomatischen Diagramme der Gegenwart hat, weil diese weniger hierarchische Vorstellungen menschlicher Vielfalt nahelegen als es das Bild des Baumes tut, macht sie daher deutlich, dass auch diese als Teil der von ihr rekonstruierten Geschichte betrachtet werden müssen. Das Buch bietet insgesamt einen spannenden Einblick in die Geschichte der diagrammatischen Visualisierungen von „Rasse“, bleibt allerdings in weiten Teilen einer oft additiv erscheinenden Darstellung der Positionen und Diagramme einzelner Forscher:innen beziehungsweise Forschungszusammenhänge verhaftet.

## Kontinuitäten, Brüche, (Un-)Gleichzeitigkeiten – Geschichte und Kritik der Gegenwart

Wissenschafts- und Wissensgeschichtsschreibung konstruieren die Gegenstände, die sie behandeln, immer ausgehend von einer Gegenwart, in der sie selbst situiert sind. Die vier vorliegenden Arbeiten gehen unterschiedlich mit dieser Problematik um. Mackenzie Cooley deutet die Relevanz ihrer Analyse für Aktualisierungen eugenischen Denkens in der Gegenwart nur an. Sie konzentriert sich auf parallele Fallstudien, wobei es ihr durchaus gelingt, überraschende Bezüge zwischen den unterschiedlichen „Stimmen“ – das heißt den mehr oder weniger gleichzeitigen „discourses of *razza*, human race, and taxonomization as well as divergences in European and American unterstandings of animal difference“ (4) – aufzuzeigen, ohne sie einer großen Erzählung zu subsumieren. Der Fokus auf die irreduziblen, multiplen Ursprünge der Begriffe *razza *und *Rasse/race*, deren Bedeutungen sich im 16. Jahrhundert zwar deutlich voneinander unterscheiden, teils aber auch überlagern, ist mit Sicherheit eine der Stärken dieses Buches, das bisher wenig bearbeitete Aspekte der Geschichte des Rassendiskurses und der proto-eugenischen Züchtungsvorstellungen der Frühen Neuzeit beleuchtet, sich aber einer Systematisierung der Befunde weitgehend enthält.

William Max Nelson, der ebenfalls den Begriff der Eugenik nutzt und historisch entgrenzt – das heißt auch auf Vorstellungen und Praktiken vor dem späten 19. Jahrhundert bezieht, als Francis Galton den Begriff prägte und sich entsprechende bevölkerungspolitische Projekte im Kontext der modernen Nationalstaaten formierten –, vertritt hingegen eine starke Kontinuitätsthese. Ihm zufolge entstanden in der Aufklärung Formen biopolitischer Intervention, „that only became more significant with the continued development of racism and eugenics in the nineteenth, the genocide and genetics in the twentieth century and the manifold possibilities of genetic engineering that have emerged in the twenty-first century“ (17f.). Die ideengeschichtliche Ausrichtung seiner Untersuchung, die sich auf die Rekonstruktion von theoretischen Positionen und politisch-konzeptionellen Vorschlägen einzelner Autoren konzentriert, stützt diese lineare Geschichtskonstruktion. Die einzelnen Kapitel des Buches bringen differenzierte und erhellende Analysen zu bevölkerungspolitischen Züchtungsprojekten im Kontext der französischen Aufklärung und insbesondere in den französischen Kolonien. Irritierend ist jedoch, dass hier – im Gegensatz zu Foucault selbst und dessen eher tentativem Umgang mit dem Begriff der Biopolitik – eine Ideengeschichte „der Aufklärung“ geschrieben wird. Diese mündet in einen erneuten Appell, den Doppelcharakter der Aufklärung als Herrschafts- und Befreiungsprojekt anzuerkennen, mit deren „Vermächtnis“ wir noch immer ringen (226). Da das „unterworfene Wissen“ und die marginalisierten, insbesondere Schwarzen und weiblichen Stimmen der Aufklärung bei Nelson aber keinerlei Aufnahme finden, bleibt sein lineares Narrativ vor allem dem Herrschaftsaspekt verpflichtet.

In anderer Weise knüpft Engelstein an Foucault an. Ihr Fokus liegt auf synchronen Entwicklungen in den sich überkreuzenden Wissensfeldern der Naturforschung/Biologie, Literatur und Sprachwissenschaft. Über Diskurse und Disziplingrenzen hinweg geht sie der Frage nach, wie die Figur des/der Geschwister immer wieder eingesetzt wurde, um Zugehörigkeit und Nicht-Zugehörigkeit, Einschlüsse und Ausschlüsse zu konstruieren. Die These, dass wir uns noch immer im Rahmen der modernen Episteme bewegen, mündet jedoch nicht in die Annahme einer ungebrochenen Kontinuität. Sie ist bei Engelstein vielmehr mit der These verbunden, dass die Geschwister-Logik heterogene Konstellationen geprägt hat und ein dezidiert kritisches Potenzial enthält, welches auf die Möglichkeit verweist, exklusive und „konfrontative Formulierungen von Zugehörigkeit, Identität und Handlungsmacht“ (3) zu überwinden.

Marianne Sommers „Diagrammatik“ der unterschiedlichen Konstruktionen von menschlicher Vielfalt in der physischen Anthropologie, der Evolutionstheorie und der modernen Genetik im Zeitraum von 1770 bis etwa 2020 ordnet das Untersuchungsmaterial zwar wiederum chronologisch auf einer linearen Zeitachse an. Indem sie die Diagramme als „performativ“ (16) begreift, gelingt es ihr jedoch – jenseits der Darstellung von Kontinuitäten und Reprisen –, die jeweils spezifischen epistemischen und politisch-ethischen Einsätze herauszuarbeiten. Die Geschichte der diagrammförmigen Visualisierungen von „Rasse“ erscheint so als ein umkämpftes Terrain, das seit dem späten 18. Jahrhundert immer wieder eng mit rassistischen, kolonialen und genozidalen Politiken sowie kritischen Gegenpositionen verbunden war. Gleichzeitig deckt auch Sommer, ähnlich wie Engelstein, ein ihrem Gegenstand immanentes kritisches Potenzial auf, das es zu nutzen gilt. Denn sofern Diagramme die Phänomene, die sie darstellen, nicht repräsentieren, verweisen sie Sommer zufolge immer schon auf ihren eigenen „(re)präsentionalen Charakter“ (293). Ihre Bildlichkeit eröffnet somit auch die Möglichkeit, die Welt anders zu sehen und zu gestalten.

## Schluss

Die vier hier besprochenen Bücher leisten interessante und wichtige Beiträge zur Erforschung der Genese und Entwicklung des modernen Rassendiskurses seit der Neuzeit. Instruktiv ist dabei sowohl die Einbeziehung von Texten und Materialien aus den nicht-europäischen Kontexten der transatlantischen – spanischen und französischen – Kolonien als auch die Erweiterung der wissenschaftshistorischen Forschung durch Analyse von Visualisierungstechniken sowie literarischem Material und Texten der politischen Ideengeschichte. Eine Gefahr solcher Erweiterungen ist zwar, dass Bezüge zwischen den einzelnen Materialanalysen und spezifischen Konstellationen vage bleiben – das heißt Befunde entweder unverbunden nebeneinanderstehen oder unter einem übergeordneten Narrativ subsumiert werden. Die Autor:innen der hier vorgestellten Bücher tendieren mal in die eine, mal in die andere Richtung. Sie machen jedoch allesamt deutlich, dass die Geschichte des modernen Rassendiskurses sich ohne solche Horizontweitungen, die durch die Erweiterung der Wissenschaftsgeschichte zur Wissensgeschichte und die zunehmende Einbeziehung postkolonialer Perspektiven in den vergangenen Jahrzehnten entwickelt haben, kaum angemessen schreiben lässt. Für die weitere Forschung wünschenswert ist darüber hinaus eine stärkere Berücksichtigung der geschlechtertheoretischen und -politischen Aspekte der Thematik und entsprechender Forschungen. Unter den hier besprochenen Büchern bearbeitet allein Engelstein die Verschränkung des modernen Rassendiskurses mit sexuellen Phantasmen und Geschlechterverhältnissen, obwohl insbesondere die Forschungen zu Projekten der Menschenzüchtung sowie Konstruktionen von Abstammung und Verwandtschaft Ansatzpunkte für eine weitergehende Analyse der geschlechtlichen Implikationen von Projekten der selektiven Reproduktion bieten.

## Data Availability

Für den vorliegenden Beitrag wurden keine Daten erhoben.
